# White Matter Reorganization and Functional Response after Focal Cerebral Ischemia in the Rat

**DOI:** 10.1371/journal.pone.0045629

**Published:** 2012-09-18

**Authors:** Chrystelle Po, Daniel Kalthoff, Young Beom Kim, Melanie Nelles, Mathias Hoehn

**Affiliations:** In-vivo-NMR Laboratory, Max Planck Institute for Neurological Research, Cologne, Germany; Julius-Maximilians-Universität Würzburg, Germany

## Abstract

After stroke, the brain has shown to be able to achieve spontaneous functional recovery despite severe cerebral damage. This phenomenon is poorly understood. To address this issue, focal transient ischemia was induced by 60 min middle cerebral artery occlusion in Wistar rats. The evolution of stroke was followed using two magnetic resonance imaging modalities: diffusion spectrum imaging (acquired before, one and four weeks after stroke) and functional magnetic resonance imaging (acquired before and five weeks after stroke). To confirm the imaging observations, immunohistochemical staining for myelin, astrocytes and macrophages/microglia was added. At four weeks after stroke, a focal alteration of the diffusion anisotropy was observed between the ipsilesional ventricle and the lesion area. Using tractography this perturbation was identified as reorganization of the ipsilesional internal capsule. Functional imaging at five weeks after ischemia demonstrated activation of the primary sensorimotor cortex in both hemispheres in all rats except one animal lacking a functional response in the ipsilesional cortex. Furthermore, fiber tracking showed a transhemispheric fiber connection through the corpus callosum, which-in the rat without functional recovery-was lost. Our study shows the influence of the internal capsule reorganization, combined with inter-hemispheric connections though the corpus callosum, on the functional activation of the brain from stroke. In conclusion, tractography opens a new door to non-invasively investigate the structural correlates of lack of functional recovery after stroke.

## Introduction

In the Western civilization, stroke is a leading cause of mortality and significant morbidity, leaving many survivors permanently disabled. Yet, the brain appears to be able to compensate the tissue damage induced by stroke and to achieve partial or sometimes even complete functional recovery [1], [2]. Thus, functional and structural brain reorganization has been observed in human and animal studies [1], [3]. During a chronic phase after stroke, changes in long-term potentiation (LTP), axonal regeneration as well as sprouting and synaptogenesis can induce cerebral reorganization [1]. This cerebral reorganization may well be the origin of the functional recovery. Therefore, it is necessary to understand the effect of the tissue changes on the functional recovery in order to design and optimize future treatment strategies. Several MRI studies in adult rats have shown that spontaneous functional recovery can be observed in the ipsilateral sensorimotor cortex after stroke [4], [5], [6]. An adaptation of the neuronal networks, possibly combined with structural rearrangements, must be considered the basis of this functional recovery.

Diffusion tensor imaging (DTI) has been used to observe structural cerebral changes in several studies in humans and in small animals. This method has become an attractive technique for the study of cerebral pathologies, in particular for stroke [7], [8]. In fact, DTI permits to determine the fiber orientations in a voxel of cerebral tissue, based on the anisotropy of the apparent diffusion coefficient (ADC) of the water [9], [10], [11]. However, DTI cannot resolve the fiber orientation in voxels containing more than one principal direction such as in the case of crossing or branching fiber tracks or in the case of partial volume effects when different types of tissue architecture are included in the same voxel [12]. To resolve this problem, diffusion spectrum imaging (DSI) was developed to extend access to complex tissue architectures [12], [13]. This method is based on the classical formalism of the “q-space” theory to recover a 3D diffusion propagator, or diffusion spectrum in each voxel. The result is presented by the orientation distribution function (ODF) of the water diffusion permitting to estimate the generalized fractional anisotropy (gFA), a conceptual extension of the fractional anisotropy (FA) determined with DTI [14].

In the present study, we report the sensitivity of the generalized fractional anisotropy to discriminate tissue areas of increased myelination at the border of the ischemic lesion. Using fiber tracking with DSI, we further demonstrate a remodeling of the internal capsule after edema formation. Finally, from our longitudinal study on rats extending over 5 weeks after stroke induction, we observe a correlation between the lack of functional recovery detected with fMRI and the structural reorganisation observed with DSI and confirmed by histology.

## Materials and Methods

### Animals

All experiments were conducted according to the guidelines laid out in the German Animal Welfare Act and approved by the local authorities (Landesamt für Natur, Umwelt und Verbraucherschutz Nordrhein-Westfalen) under permission number 9.93.2.10.31.07.048.

19 male Wistar rats (Janvier, France) of bodyweight 365±25 g were used. Animals were kept in cages at a 12/12 h light/dark cycle and had *ad libitum* access to water and standard diet (15 g/day/rat). Animals were housed individually to assure that each rat had access to the same amount of food for the dietary purpose.

### Ischemic stroke model

The ischemic lesion was induced in 15 rats by transient (60 minutes) occlusion of the right middle cerebral artery (MCA), using the modified intraluminal filament technique [15], [16] under 2% isoflurane anesthesia in 30/70% O_2_/N_2_O. The body core temperature was measured with a rectal temperature probe and maintained at 36.7±1°C using an automated heating blanket with temperature feedback (Medres, Cologne, Germany). The occlusion period and the successful reperfusion of the right MCA were controlled by continuous recording of ipsilateral laser-Doppler flowmetry (LDF), with a flow decrease of around 75% observed during the ischemic period. Animals were excluded from the study if they did not show a lesion in the caudate putamen or in the caudate putamen and the cortex on T_2_-weighted MR images (T2WI) at 48 h after surgery. Four separate animals served as healthy control and did not undergo surgery. These healthy animals permitted to control the quality and reproducibility of our post-processing protocol. We have chosen healthy animals instead of sham animals to void any interference, even from sham surgery with the fiber patterns, but rather to obtain a reliable picture of the situation in healthy, intact animals.

### Experimental protocols

For MRI experiments, anesthesia was induced with 2.0–2.5% isoflurane. Then, animals were fixed in an animal cradle (Bruker BioSpin, Ettlingen, Germany) using a toothbar and earbars for stable positioning of the head. During MRI experiments, the anesthesia was maintained with 1% isoflurane, except for the fMRI study (cf below). Respiration was monitored using a pressure-pad under the thorax. Blood oxygen saturation and cardiac cycle were measured with a pulse oxymeter (SA Instruments Inc., NY, USA) attached to the hindpaw (for fMRI only). The body core temperature was monitored using a fiber optic rectal temperature probe and controlled with an automated temperature control unit (medres, Cologne) employing a water blanket.

To minimize the anesthesia period and the concomitant stress on the animals by the *in vivo* experimentation, MRI acquisition time was limited to approximately 2 hours. Because of this time limitation and the different anesthesia protocol conditions, DSI and fMRI experiments were recorded during separate imaging sessions. DSI experiments were acquired at two weeks before, and one and four weeks after stroke, while fMRI experiments were acquired at one week before and five weeks after stroke.

### In vivo MR Imaging

T_2_-weighted MRI (T2WI) for lesion screening was recorded at 2 days after stroke, on a 4.7 T BioSpec system with 30 cm bore size (Bruker BioSpin, Ettlingen, Germany), equipped with 100 mT/m gradient sets and operating under PARAVISION 4.0 software (Bruker BioSpin). Here, a 120 mm Helmholtz coil was used for RF transmission, combined with a 25 mm diameter surface coil for signal detection.

DSI and fMRI experiments were conducted on a 11.7 T BioSpec system with 16 cm bore size (Bruker BioSpin), equipped with 750 mT/m gradient sets and operating under PARAVISION 5.0 software (Bruker BioSpin). Transmission was achieved with a quadrature volume resonator (inner diameter 72 mm) while a rat brain quadrature surface coil (30×30 mm^2^) was used for signal reception (Bruker BioSpin).

#### T2WI protocol

The localization and severity of the infarct were observed with T2WI at 4.7 T, at two days after stroke. T2W images were obtained using a spin echo (SE) sequence with the following parameters: TR/TE = 4200/69.5 ms, field of view  =  30×30 mm^2^, matrix  =  192×192, 27 contiguous slices with a thickness of 0.5 mm, signal average of 2, acquisition time, TA = 13 min 26 s.

To provide a common reference across all sessions at 11.7 T, T_2_-weighted anatomical images were acquired using a turbo rapid acquisition with relaxation enhancement (TurboRARE) sequence with the following parameters: TR/TE = 4000/32.5 ms, RARE factor  =  8, field of view  =  32×32 mm^2^, matrix  =  256×256, 28 contiguous slices with a thickness of 0.5 mm, and TA = 4 min 16 s.

#### fMRI protocol

The experimental protocol for the fMRI anesthesia followed an earlier developed and applied protocol, as described elsewhere [6], [17], [18], [19]. Briefly, the insertion of the stimulation needle electrodes into the forepaws and the subcutaneous needle for infusions, as well as the initial pilot scans and adjustments were performed under 1.5–2.0% isoflurane in 30/70% O_2_/N_2_O. Then, a bolus of 0.5 ml Medetomidine solution (Domitor^TM^, Pfizer; 1 ml/kg bodyweight, added to 10 ml saline solution) was injected through the subcutaneous needle, then isoflurane was slowly discontinued to zero and N_2_O exchanged for N_2_. To allow for stabilization of physiological conditions, fMRI acquisition and continuous infusion of Medetomidine solution (1 ml/h) were not started until 20 minutes after bolus injection of Medetomidine. After completion of the fMRI experiments, the Medetomidine antidote Atipamezole (Antisedan, Pfizer, 1 ml/kg bodyweight) was injected subcutaneously with 2 ml of saline solution to reverse the sedative effect and to substitute for fluid loss during the experiment.

Electrical forepaw stimulation was performed applying rectangular pulses (1.5 mA, 6 Hz, 0.3 ms pulse width; home-built stimulation unit) to the two electrodes inserted under the skin in each paw. The paradigm consisted of five blocks (1 block: 45 s resting period and 15 s activation period) and ended with an additional 45 s resting period, resulting in 115 image repetitions in a total experimental time of 5 min 45 s. A single-shot gradient-echo EPI sequence was used with the following parameters: TR/TE = 2840/17.5 ms, field of view  =  28.8×28.8 mm^2^ with 300 µm in-plane resolution, 5 contiguous slices with 1.2 mm slice thickness, centered at the somatosensory cortex. Within one anesthesia session, this series of acquisitions was repeated 3 times for each paw in an alternating fashion with approximately 30 min. in between.

#### Diffusion Spectrum Imaging (DSI) protocol

During the DSI sessions on the 11.7 T, anesthesia was maintained with ∼1.5% isoflurane in 30/70% O_2_/N_2_O. For the DSI data, we applied 203 diffusion gradient vectors, corresponding to a set of isotropically distributed 3D grid points in q-space, as reported earlier [20]. In short, twelve subsets for a complete scan were acquired using a diffusion tensor spin-echo echo-planar imaging (DTI-SE-EPI) sequence with the following parameters: four shots, two repetitions, TR/TE = 3000/37.9 ms, 20 consecutive slices with resolution  =  0.2×0.2×0.5 mm^3^, Δ/δ = 25/5 ms. The b-values and the number of diffusion directions for q-space encoding for the twelve subsets were as follows: 154/6, 308/12, 462/8, 615/6, 769/24, 923/24, 1231/12, 1384/30, 1538/24, 1692/24, 1846/8, 2000/24 (s/mm^2^/# of directions). Experimental scan time for one complete DSI data set, including two averages, was 102 min.

### Histology

After the last MRI measurement, the rats were sacrificed under deep isoflurane anesthesia by transcardial perfusion-fixation with 0.1 M phosphate-buffered saline, followed by 4% para-formaldehyde in phosphate buffer. The brains were removed, immersed overnight in the same fixative, and transferred to cryoprotective sucrose solution (30%) for at least 3 days.

For anatomic presentation, tissue sections were stained with haematoxylin and eosin (HE).

Glial fibrillary acidic protein (GFAP) is an intermediate filament upregulated in response to injury to the central nervous system and was used as a marker for astroglial reactivity in the brain. Immunohistochemistry was performed to stain for neurons, astrocytes and macrophages/microglia. Primary antibodies were diluted as follows: Mouse anti rat CD68, (Serotec) 1∶2000, mouse anti-NeuN (Chemicon MAB 377) 1∶500 and rabbit anti-GFAP (Dako) 1∶500. Second antibodies used were: Cy-3 goat anti mouse (1∶500; Jackson Immunoresearch,) and biotinylated goat anti rabbit (1∶500, Vector). The Hoechst dye (1∶1000) was used for visualization of primary antibody binding. Alternatively, stain for macrophages/microglia, the monoclonal antibody ED-1 (Serotec, Oxford, UK), which recognizes a lysosomal antigen on both phagocytic microglia and blood-borne macrophages, was used at a dilution of 1∶2000. The Vectastatin avidin-biotinperoxidase method (Vector Labs, Burlinghame, CA, USA) and 3,3-diaminobenzidine (DAB) were used to visualize the primary antibody binding. Then slices were mounted and counterstained with cresyl violet.

For myelin presentation, sections were stained with Luxol Fast Blue solution, washed in 95% alcohol, and then placed in 0.05% lithium carbonate. After a second washing in 70% alcohol and distilled water, sections were counterstained with 0.1% Nuclear Fast Red.

The sections were analyzed and digitally photographed using a Leica MZ FL III microscope (Leica, Bensheim, Germany), equipped with a CCD camera.

### Image post-processing

#### BOLD fMRI analysis

First, fMRI datasets were converted to analyze format and underwent motion correction (mcflirt, FMRIB Software Library, http://www.fmrib.ox.ac.uk/fsl), performed separately for each slice and restricted to in-plane translations and rotations. From those datasets, with temporal mean and maps of the temporal SNR, BOLD signal change and p-value were calculated on a voxelwise basis. Significance level was determined by a t-test between the rest and stimulation periods. Thus, all image based processing was performed with ImageJ (Version 1.42q; National Institutes of Health, Bethesda, USA; http://rsbweb.nih.gov/ij) using custom-made plug-ins and macros that utilize the Apache Commons Maths Library (Version 2.1; The Apache Software Foundation; http://www.apache.org) [21]. BOLD signal maps were color-coded and overlaid on the corresponding mean signal intensity EPI images for visual presentation.

### DSI reconstruction and tractography

To reduce the image drift induced by the increasing gradient heating due to the extreme duty cycle [21] and the interference between echo and diffusion gradient pulse [22], rigorous image processing was applied, as previously described in detail [20]. After the co-registration of all individual images, we used DSI Studio (dsi-studio.labsolver.org) to reconstruct DSI data, to calculate gFA maps (generalized fractional anisotropy), and to perform tractography [13].

As an extension of the FA metric, defined as: 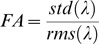
where λ are the eigenvalues of the diffusion tensor, we defined the gFA:
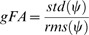


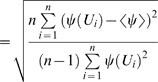
where s.d. is the standard deviation and r.m.s. is the root mean square, <ψ> is the mean of the ODF.

gFA as well as FA is comprised between 0 and 1.

The localization of the gFA alterations on the ischemic hemisphere was determined on gFA maps obtained at 4 weeks after stroke. After conversion to analyze format, the FMRIB's Automated Segmentation Tool (FAST, FMRIB Software Library) was used to segment this perturbation area based on a hidden Markov random field model and an associated Expectation-Maximization algorithm. Then, MRIcro 1.4 software (www.mricro.com) permitted to determine the volume and to save it as mask in image format compatible with DSIstudio software (dsi-studio.labsolver.org) [23], [24]. This region of interest on the ipsilesional hemisphere (ROI_ipsi_) was used for further analysis. A corresponding homotopic ROI on the contralesional hemisphere was defined (ROI_contra_). Maps of gFA acquired before stroke were co-registered, based on anatomical reference data, to the gFA maps obtained at 4 weeks after stroke to assure identical ROI selections. Following the same procedure, the infarct volume was determined from the T2W images.

Tractography was performed based on a streamline tracking method with the computation terminated after 50.000 seeds using the following parameters: gFA threshold  =  0.2, turning angle  =  63. Fiber-tracking was generated using two different seed regions:

- To observe the white matter organization, the medial portion of the corpus callosum between 1.6 mm rostral to bregma and 0.4 mm caudal to bregma, according to the rat brain atlas [25], was used as seed area.
- To characterize the area of gFA alteration, previously determined, this zone was chosen as seed region.

The determination of fibers was terminated when one of the two thresholds (gFA = 0.2; turning angle  =  63°) was reached or when the fiber tract reached a voxel without signal or fiber (as is the case at the inner skull surface).

### Statistical analysis

The statistical analysis of the gFA data was performed by one way ANOVA and multiple comparison procedures (Holm-Sidak method). A value of p<0.05 was considered statistically significant. Calculations were performed with a standard statistical software package (SigmaStat 3.1, Systat software). All results are presented as mean value±SEM.

## Results

### Stroke lesion

**Figure 1:**
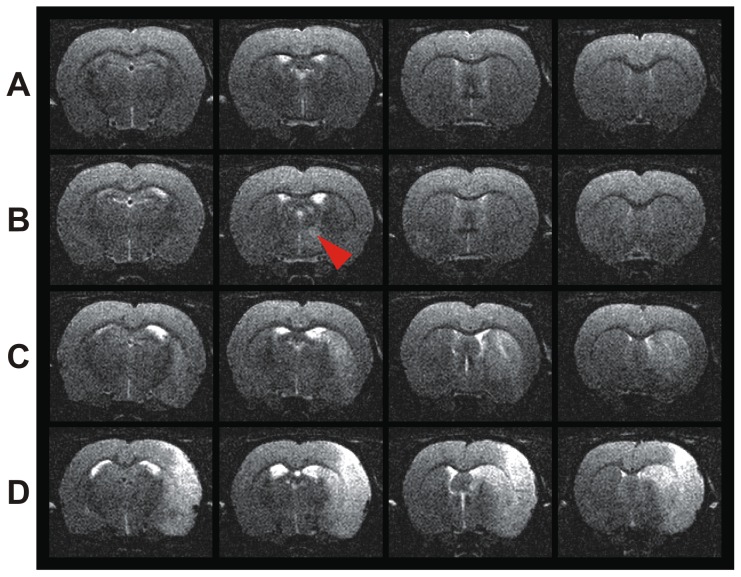
T_2_-weighted coronal images for the evaluation of ischemic territory. Consecutive T_2_-weighted coronal slices (thickness  =  0.5 mm) obtained at two days after stroke at 4.7 T in four representative rats: one rat without edema (A), one with an edema in amygdala (red arrow) (B), one with edema in the caudate putamen (C), and one with edema in the caudate putamen and the cerebral cortex (D).

Four rats were excluded from subsequent experiments, one based on lack of lesion detection on T2WI at 48 h after stroke induction (Fig. 1A) while the other three presented a lesion only in the amygdala (Fig. 1B). In animals with successful stroke induction (n = 11), hyperintensity was observed on T2WI in the caudate putamen in eight rats (Fig. 1C) and in both, caudate putamen and cortical tissue in three further animals (Fig. 1D).

### fMRI

**Figure 2:**
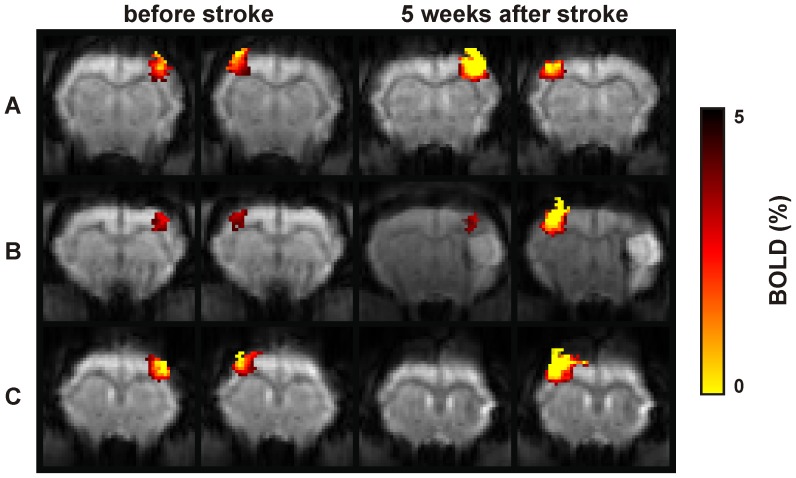
Forepaw stimulated activation of the somatosensory cortex before and after stroke. Positive BOLD response observed at 11.7T during forepaw stimulation of the left (first and third column) and right (second and fourth column) paw in three rats (A–C) before, and at five weeks after stroke. Rat A is characterized by a stroke localized in the caudate putamen, rats B and C are characterized by a stroke localized in caudate putamen and cerebral cortex. Before, and five weeks after stroke, a positive BOLD signal was observed in S1 cortex ipsi- and contralateral to the lesion. Just in one rat (C), absence of a significant BOLD response was observed in the ipsilesional S1 cortex.

Before stroke induction, all animals showed activation of the forelimb region in the somatosensory cortex (S1fl) contralateral to the stimulated paw, as expected (Fig. 2). Five weeks after stroke, the activation remained unchanged in the contralesional, healthy hemisphere. Ten out of eleven rats also presented S1fl activation in the ischemic (right) hemisphere upon left forepaw stimulation. Only in one animal with cortico-striatal ischemic lesion, no significant BOLD signal was detected in the ipsilesional S1fl cortex at 5 weeks after stroke, upon stimulation of the left paw (Fig. 2C).

### gFA map

**Figure 3:**
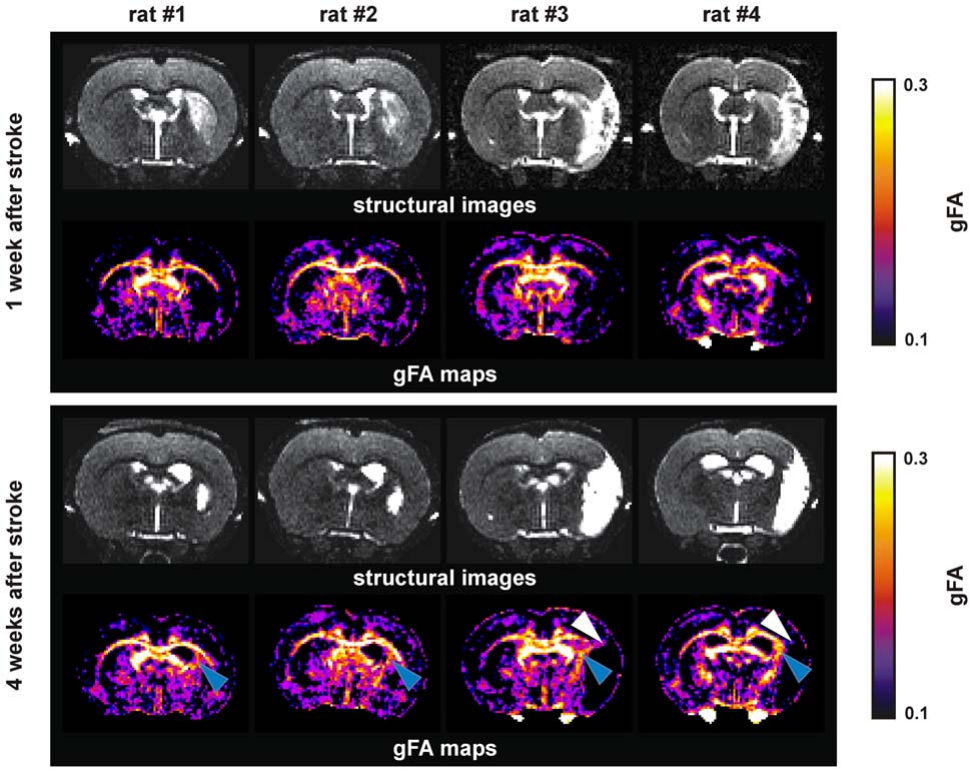
Detection of diffusion anomaly on fractional anisotropy maps after stroke induction. T_2_-weighted images (upper row of each tableau) and gFA maps (lower row of each tableau) are presented at one and four weeks after stroke in four different rats. At four weeks after stroke, in each animal, a perturbation of the gFA between the stroke area and the ventricle was observed (blue arrows). In rats characterized with stroke in the caudate putamen and cerebral cortex, an increase of the gFA was detected, localized between the infarcted lateral cortex area and the healthy somatosensory cortex S1 (white arrows) at four weeks after stroke.

**Figure 4:**
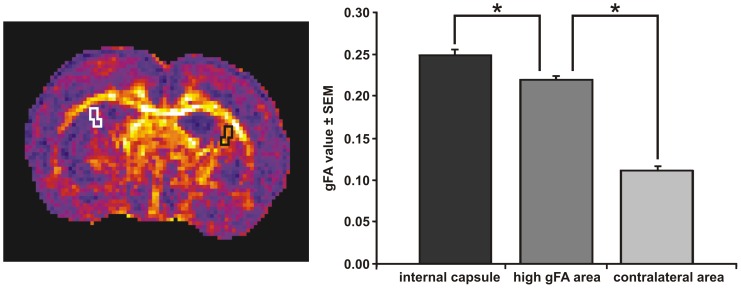
Quantification of gFA changes after stroke. At four weeks after stroke, gFA values were estimated in the contralateral internal capsule and in the high gFA area at the ischemic side (black ROIs on the gFA map), and in the corresponding contralateral area (white ROIs on the gFA map). The graph indicates the gFA value of normal internal capsule, being significantly higher still than the elevated gFA value of the ipsilateral area with increased gFA between lateral ventricle and cortex. *: p<0.05 compared to the gFA estimated in the high gFA area.

At 4 weeks after stroke, an area characterized by a high gFA value (ROI_ipsi_) was observed in all ischemic animals except one rat that had a very small infarct in the caudate putamen. This area of high gFA was localized between the ipsilesional lateral ventricle and the ischemic territory (blue arrows in Fig. 3) in the caudate putamen. The gFA values obtained before stroke, in the homotopic ROIs of both hemispheres (ipsilesional: 0.11±0.009; contralesional: 0.12±0.009) were similar to the value measured in ROI_contra_ at 4 weeks after stroke (0.11±0.006). At four weeks on the ipsilesional hemisphere, however, the gFA value in ROI_ipsi_ (0.22±0.006) showed a significant increase in tissue anisotropy (*F*(1.57) = 17.8, p<0.05) compared to the stable value in ROI_contra_, unaffected by the stroke induction. This gFA increase was not noticeable in the early subacute time point, one week after stroke onset. In order to verify the myelin composition of the tissue in the ROI_ipsi_ , the gFA in the healthy internal capsule (in healthy contralesional hemisphere of ischemic rats) was measured and taken as white matter reference. This gFA value (0.25±0.005) was significantly higher (*F*(1.57) = 3.25, p<0.05) than the one in the ROI_ipsi_ (Fig. 4).

### Fiber tracking

**Figure 5:**
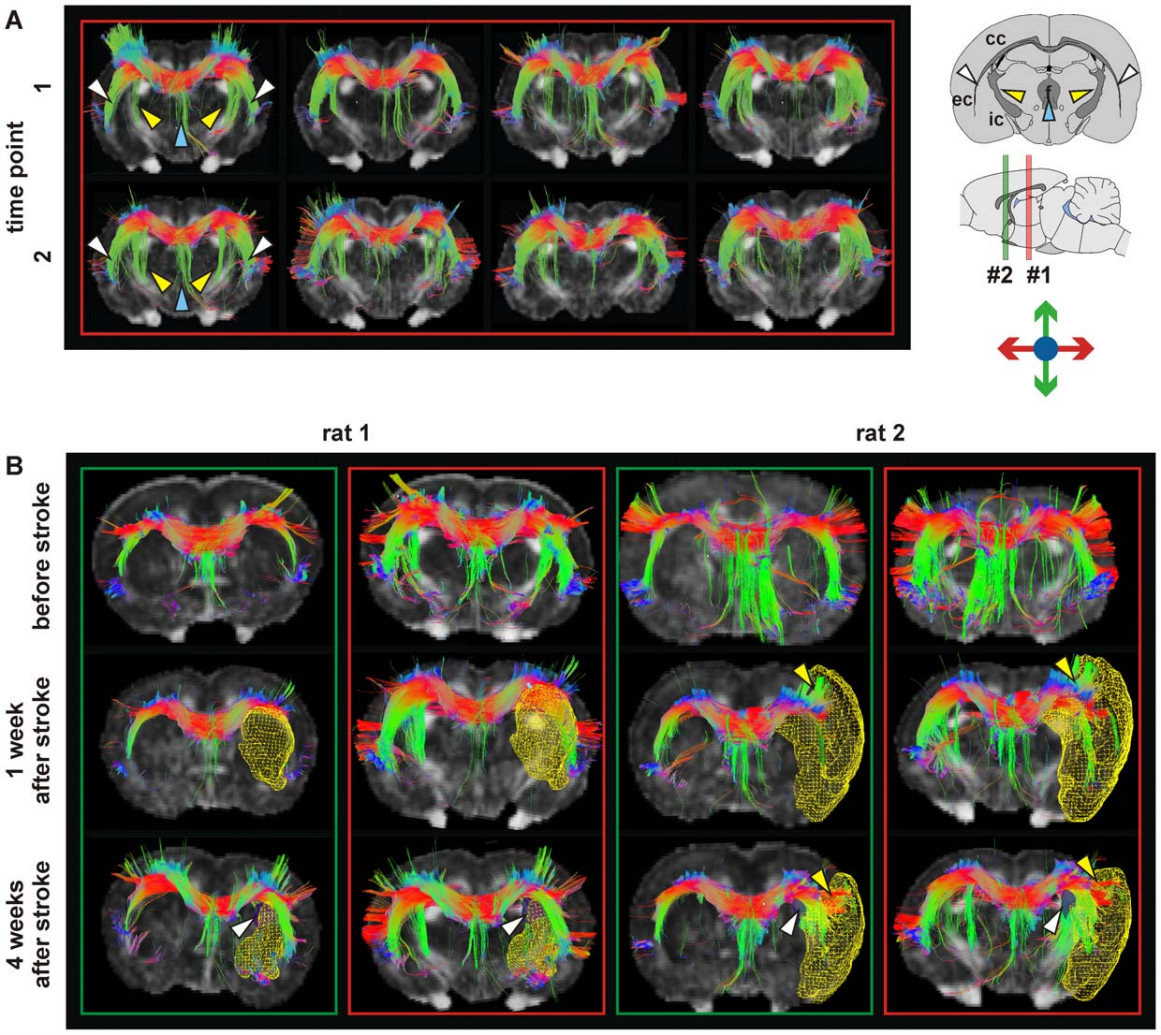
Tractography in healthy and ischemic conditions. Tractography of four healthy rat brains (four columns) is performed with corpus callosum as seeding area at two time points (t1, t2) four weeks apart (Panel A). In all cases, the fibers of the corpus callosum followed the white matter going through the internal capsule (yellow arrows), external capsule (white arrows) and fornix (blue arrows). In panel B, tractography in two rats before stroke and one week and four weeks after stroke is depicted. In the two green boxed rows, the reference slice (a gFA map) is close to bregma corresponding to position #2 on the sagittal scheme (green line) whereas in the two red boxed rows, the reference slice is more caudal as indicated by position #1 on the scheme (red line). Lesion volumes, determined from T2WI, are depicted as a yellow mesh. Rat 1 developed a stroke in the caudate putamen, and rat 2 in the caudate putamen and cerebral cortex. The fiber-tracking representation permitted to observe, on the one hand, at 4 weeks after stroke, a modification of the connection between corpus callosum and the internal capsule (white arrows). On the other hand, from one week after stroke, in rats with stroke in caudate-putamen and cerebral cortex (rat 2), an extension of the corpus callosum into the cerebral cortex separating the infarct area and the rest of the cortex (yellow arrows) was observed. The figures show color-coded tracts in the horizontal (red), vertical (green) and transversal (blue) directions, as indicated in the arrow schematic at top right.

In the four healthy control rats, tractography with corpus callosum as seed region (see Figure 5 A) showed that the fibers from the corpus callosum follow the white matter going through the internal capsule (yellow arrows), external capsule (white arrows) and fornix (light blue arrows). Stability and reproducibility of the fiber patterns were demonstrated by comparison of the same healthy animals across two independent time points (2 weeks apart), and across several healthy rats (n = 4) at one given time point.

Applying the same fiber-tracking parameters, the connection between corpus callosum and internal capsule was assessed before stroke, and at 1 and 4 weeks after stroke. Compared to the fiber tracks in healthy animals and to the contralesional healthy hemisphere in ischemic animals, this connection shifted to a more frontal position at 4 weeks after stroke (Fig 5 B: white arrows). These fibers corresponding to the link between the corpus callosum and the internal capsule went through the region characterized by high gFA (Fig. 5 B: white arrows).

Fiber tracts in three rats with stroke in the cortex and caudate putamen showed a reduction of the density of fibers in the ipsilesional external capsule compared to the contralateral side, both at 1 and 4 weeks after stroke. Further, a set of fibers leaving the corpus callosum to enter into the cortex was observed, separating the cortical infarct area and the rest of the cortex (Fig. 5 B: yellow arrows). At 4 weeks after stroke, this extension of fibers correlated with the local increase of gFA within the maps (Fig 3 white arrows) while at 1 week after stroke, such gFA changes were not yet detectable.

**Figure 6:**
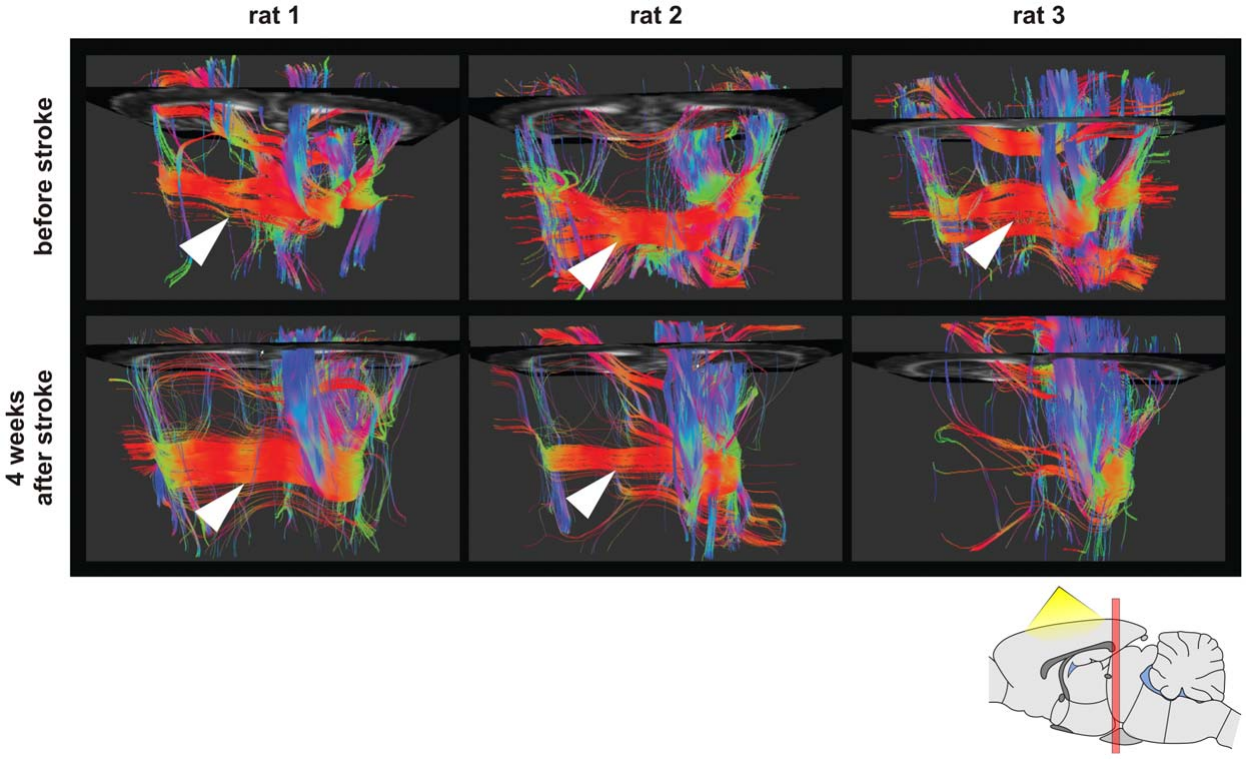
Fiber-tracking results obtained with the area of high gFA chosen as seed region. The view on the determined fiber bundles is presented from above the brain (see scheme). Results of three rats are displayed before and four weeks after stroke, respectively (rat 1: stroke in the caudate putamen; rat 2 and 3 s stroke in the caudate putamen and cerebral cortex). Interhemispheric connection through callosal tracts is clearly observed before and after stroke in all cases (white arrows), except for rat 3 which did not show functional recovery on fMRI data.

In order to characterize the gFA perturbations localized between the ventricle and the infarct area, this region (ROI_ipsi_) was used as the seed area in a second tracking analysis. The tractography permitted to observe in all ischemic rats a connection of this area with the contralateral hemisphere via the corpus callosum (Fig 6: white arrows). However, in the rat which did not present functional activation at 5 weeks after stroke, this transhemispheric fiber connection was lost (Fig. 6, Rat #3).

### Histology

**Figure 7:**
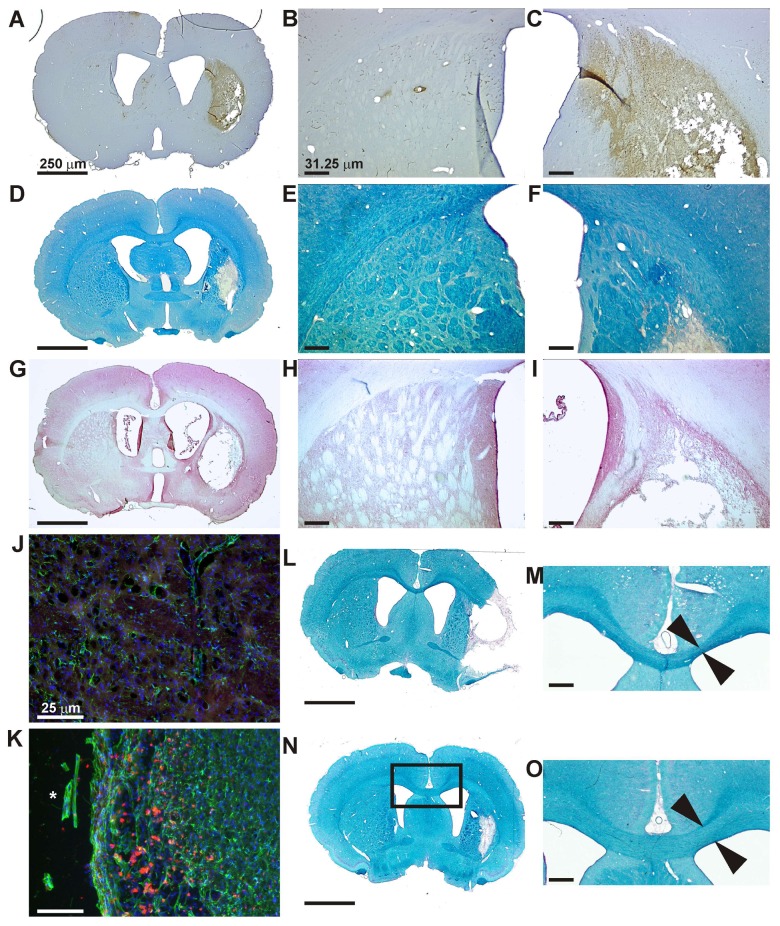
Histological sections obtained at five weeks after stroke and stained for myelination and inflammation. ED-1 combined with cresyl violet (A, B, C) shows the presence of macrophage/microglia around the infarct area and in particular between the ventricle and lesion (C); no ED-1 positive cells were observed in the contralesional hemisphere (B). Luxol fast blue (D, E, F) and hematoxylin eosin (G, H, I) staining show the presence of myelin between the ventricle and the infarct (F and I) in contrast to the contralateral side (E and H). GFAP (in green) and CD68 (in red) permitted to detect activated astrocytes and macrophages/microglia, respectively (K) in the border of the lesion area (the asterisk indicates the lesion territory) of the cortex compared to the corresponding contralateral area (J). Comparison of the thickness of the corpus callosum on the ipsilateral and contralteral side (L–O). The luxol fast blue staining of the animal without functional recovery on BOLD shows a distinctly thinner corpus callosum (L,M) compared to the typical situation in a representative case of the other, recovering animals (N,O). Size bar is equivalent to 250 µm in all tissue section overviews (A,D,G,L,M), is 31.25 µm in enlarged sections (B,C,E,F,H,I,M,O) and is 25 µm in the immunohistochemically stained sections (J,K).

The area, characterized by a high gFA value on the gFA maps between the ipsilesional lateral ventricle and the infarct zone, presented a high concentration of macrophages or activated microglia, detected by ED1 staining (Fig. 7 A–C). Furthermore, in the same area, an intense myelin signal was detected on Luxol fast blue stained tissue sections (Fig. 7 D–F). For comparison, hematoxylin and eosin stained adjacent sections are included (Fig. 7 G–I). Activated astrocytes and macrophages/microglia were detected in the border of the lesion area in the cortex using GFAP and CD68 staining, respectively (Fig. 7 J–K).

The thickness of the ipsilesional corpus callosum is demonstrated in Fig 7 for a representative animal showing functional response (Fig. 7 N–O). A decrease of the corpus callosum thickness (around 30%) on the ipsilesional side was observed only in the rat which did not show a positive BOLD signal at five weeks after stroke (Fig. 7 L,M).

## Discussion

To the best of our knowledge, this is the first study applying diffusion spectrum imaging to *in vivo* studies of experimental brain pathology in small animals. This was achieved in an experimental scan time compatible with the requirements to keep anesthesia periods short in order to minimize the stress on the already weakened animals during repetitive MRI sessions in a longitudinal study. Using generalized fractional anisotropy and fiber tracking, we have been able to follow the cerebral dynamics reflected by structural reorganizations during a month following stroke. Furthermore, the combination of DSI with fMRI pointed to a relationship between functional brain activation and the presence of the observed transhemispheric fiber connection.

### Tractography methodology

In general, tractography is a powerful but at the same time delicate method. In fact, the fiber-tracking may be influenced, mainly, by two external factors: the accuracy limits of the diffusion imaging to describe tissue with complex architecture as in the case of fibers crossing or branching and, secondly, the choice of the appropriate seed regions [26].

We have reduced the effect of the first factor by using DSI, which reconstructs an orientation distribution function (ODF) with high angular resolution instead of reducing diffusion information to a tensor model, as in diffusion tensor imaging (DTI). DSI has been demonstrated to be more sensitive to resolve the orientation structure of complex fiber architectures [12]. To our knowledge, this is the first DSI study on stroke animals under in vivo conditions.

Concerning the choice of appropriate seeds for tracking, two seed regions were selected, each one corresponding to a particular focus of the present investigation. In order to leave fiber directions from the seed unbiased, just one seed region was used to perform the fiber tracking without definition of target regions. In fact, observations were clearly different, selective and characteristic for the chosen seed regions: corpus callosum as seed region characterized the white matter organization in general, whereas the choice of gFA alteration area as seed region led to structural re-arrangement information specific about this zone resulting from stroke. The seed area must be selected corresponding to the question raised to obtain unambiguous interpretation of the results. It will be helpful to further validate the results with other imaging methods providing axonal tracing information such as manganese enhanced MRI (MEMRI) or, alternatively and invasively, by histology.

### Functional brain activation

We have investigated the modifications of structural brain organization, which may be linked to lack of functional recovery after an ischemic period, and, more precisely, may allow the discrimination between successful or lacking functional recovery of the S1 cortex detected with fMRI. As reported earlier [6], fMRI permits to divide stroke animals into two groups depending on successful spontaneous recovery within three weeks after stroke onset. All animals with no activation recovery of the S1 somatosensory cortex during this period were shown to lack functional recovery during the next six months [17]. We used this criterion to assess irreversible functional damage at the safe time point of 5 weeks post-stroke induction. Indeed, ten out of eleven animals presented with functional activation of the S1 cortex on the ischemic hemisphere. Only one animal with a cortico-striatal ischemic lesion did not show functional recovery of the S1 cortex at this time.

### White matter reorganization

DSI data – usually visualized using ODFs – can also be represented by the scalar measure of generalized fractional anisotropy (gFA) which is useful to generate tissue contrast. gFA is an extension of the fractional anisotropy (FA) observed with DTI [14]. The FA has been described as a marker of the degree of myelination and fiber density of white matter [11]. For this reason, FA, and thus also gFA, represents an interesting parameter to investigate in stroke studies. In the case of complex tissue architecture, the FA value obtained with DTI can be underestimated, and, thus, may introduce false interpretations [27]. The use of gFA, on the other hand, derived from DSI data, can reduce possible interpretation errors, thus allowing for a more precise description of the myelination and fiber density in complex tissue architecture.

In all rats, at 4 weeks after stroke, an area with a high gFA was observed, localized between the infarct in the caudate putamen and the ipsilesional lateral ventricle which is very close to the subventricular zone (SVZ). The area of increased gFA was characterized as white matter by myelin staining. This interpretation is supported by results previously described in the literature [4], [28], [29], [30] which identified the development of a high FA area in relation to reorganization of white matter such as axonal remodeling and remyelination. In this context, it should be noted that the identified area of high gFA is close to the SVZ, one of the two identified regions of neurogenesis in the brain. Although no data are presently available to support a relationship between neurogenesis and the area of increased gFA, it may be of interest to investigate this aspect in future studies using e.g. BrdU to identify new cells in the region.

In the present investigation, the gFA value measured in this tissue region was higher than in the contralesional area, confirming the earlier findings and interpretation [4], [28], [29] of this area as white matter. However, despite the clear indications for strong myelin components by the gFA value of this region, the gFA value was significantly lower than the gFA value in the normal, contralateral internal capsule. This may indicate the presence of another biological component different from myelin and decreasing the effective anisotropy. Based on the histological results, we assume that this other component, responsible for the lowering of gFA from the value of pure myelin, may well be the observed gliosis reaction, accompanied by the high concentration of macrophages/microglia observed in this specific area.

The fiber tracking with corpus callosum as seeding area permitted to construct a 3D representation of the fibers of the rat brain white matter. Thus, in the healthy animals, these fibers went though the corpus callosum, the internal and external capsule, and the fornix, identified by comparison with the rat brain atlas [25]. In ischemic animals, the fibers going through the area with high gFA value were observed and identified as the internal capsule. Tractography permitted to identify the white matter reorganization as a rostral displacement of the internal capsule, i.e. towards the front of the brain. This structural change, observed in all ischemic rats, is believed to be induced by the edema formation in the caudate putamen during the first post-stroke week when the edema is most strongly expressed. This edematous swelling then induced pressure on the adjacent cerebral tissue. This pressure, again, will have led to a decrease of the ipsilateral lateral ventricle; it may also have induced the structural organization change. The inflammatory response alone, demonstrated to be already pronounced at 1 week after stroke [31], cannot account for this modification on gFA detectable only after 4 weeks.

### Fiber extension from the corpus callosum into the cortex

In rats with an infarct localized in both the caudate putamen and the cerebral cortex, tractography from the corpus callosum permitted to observe a branching prolongation of the corpus callosum into the cortex, separating the ischemic lateral cortex and the healthy sensorimotor cortex. This was observed both at 1 and 4 weeks after stroke. At 4 weeks after stroke, the gFA map indicated a high gFA in the area of this prolongation, whereas the gFA map at 1 week after stroke did not yet show such gFA alteration. Thus, tractography appears to be more sensitive and more suited than gFA maps for the detection of reorganization of the cerebral tissue. Histology at 5 weeks after stroke allowed the identification that these fibers are colocalized with high density of activated astrocytes. However, although the tractography permits the visualization of cerebral connectivity, this method does not allow discrimination whether the determined connections are functional, indeed, and in which direction they are linked. For this purpose, the additional use of neuronal tracers may in the future resolve the functionality of such apparently new connections.

### White matter reorganization vs functional response

A correlation of the appearance of this white matter reorganization with behavioral recovery was previously described [28], [29]. In our study, this white matter reorganization was not always combined with a functional activation observed by fMRI. This apparent contradiction can be explained by the difference between the methods used to observe functional recovery: in previous studies behavioral tests were used such as neurological severity score, adhesive removal test and foot-fault test [28], [29], whereas fMRI employing forepaw stimulation was used in the present study. Actually, the majority of behavior tests do not allow the discrimination between a functional recovery linked to real tissue recovery and a compensatory phenomenon [32] while fMRI specifically characterizes the brain area stimulated by the selected stimulus. This, therefore, allows the determination of recovery of the original representation area. Besides, the tractography, using the high gFA area as seed region, pointed to a relationship between functional activation in S1 cortex and the interhemispheric connection passing though the corpus callosum. In this case of fiber disruption going through the corpus callosum, the myelin staining indicated shrinkage of the corpus callosum to 0.16 mm thickness compared to the thickness of 0.27 mm on the contralateral side. Therefore, the absence of interhemispheric connections demonstrated by tractography could be linked in part to the resolution limits of our images (0.2×0.2×0.5 mm^3^). The corpus callosum is the main pathway connecting the two cerebral hemispheres. Composed of complex bundles of fibers with distinct components that act separately, the function of the corpus callosum may be inhibitory or excitatory, depending on the task (for review see [33]). The possible correlation between functional recovery and interhemispheric connection was previously described [34], [35]. The mechanisms leading to functional recovery after a stroke period remain still unclear. Furthermore, our results need to be confirmed in the future by observations in more animals lacking spontaneous recovery by fMRI standards, and also, especially, by the parallel use of neuronal tracer methods such as MEMRI.

To conclude, this study was the first to use DSI to observe and characterize the white matter reorganization, and, thus, to prove a strong concordance of this data representation with basic gFA maps. Besides, the tractography appears, through our observations, to be a promising non-invasive tool to understand the structural correlates of lack of functional recovery after an ischemic period. This study showed the influence of the internal capsule reorganization combined with inter-hemispheric connections through the corpus callosum on the potential functional recovery of the brain from stroke.
